# Atropine

**Published:** 1911-05

**Authors:** George L. Servoss

**Affiliations:** Fallon, Nevada


THE
### TEXAS MEDICAL JOURNAL.
Established July, 1885

F. E. DANIEL, M. D., - - - - - - Editor, Publisher and Proprietor

Published Monthly.—Subscription $1.00 a Year.

Vol. XXVI AUSTIN, MAY, 1911. No. 11

The publisher is not responsible for the views of the contributors.

### Original Articles.

For Texas Medical Journal.

### Atropine.

BY GEORGE L. SERVOSS, M. D., FALLON, NEVADA.

It is probable that there is no active principle known which has a wider scope of action than has atropine, both good and bad. The group of plants from which atropine is obtained have been employed time out of mind for the majority of ills to which man is heir, but their application was feared because of the uncertainty of action and untoward and dangerous effects to be anticipated. Such uncertainty was due to the lack of knowledge of the chemical composition of the plants, due to differences in the growth of the plants under different conditions. A plant grown under one condition might be possessed of a high active principle, while under different condition might be low in such content, consequently the employment of the entire drug, or preparations thereof, was liable to produce unlike effects in like conditions, and not infrequently did atropine poisoning follow. With the isolation of this principle, and a thorough study of its actions, its values were recognized and, because of the certainty of action, it became available in numerous instances where fear had existed regarding the employment of the entire plants carrying it.

Atropine was discovered in 1831, and in 1833 was isolated by Geiger and Hesse. It occurs in prismatic, needle crystals, soluble in 300 parts of cold water, 58 parts of boiling water; very soluble in alcohol, in 35 parts of cold ether, 6 parts of boiling ether, 3 parts of chloroform, and to a marked extent in amylic alcohol. It is derived from belladonna, hyoscyamus, stramonium, scopola, mandragora and to a limited extent from the leaves of the potato and tobacco. While effective upon man and dogs, other animals eat the plants without any of the atropine effect. Belladonna probably gives more of the atropine effect than do any of the other plants in which it occurs.

The symptoms following the exhibition of atropine to physiologic effect are successively; dryness of the mouth and throat, thirst, disordered vision, paralysis of accommodation, alteration of the voice, aphonia, sense of cold followed by rapid pulse, redness of the face, vertigo, headache and delirium. With a discontinuance, the symptoms subside in reverse order within a few hours, with the possible exception of the ocular, which may persist for some time. Very large doses are followed by like symptoms, but accentuated, and paralysis occurs early. Following such dosage, swallowing is impossible, rabic symptoms appear, the conjunctiva is injected, the eyes are prominent and there is disappearance of the iris, the face becomes excessively red, there is furious delirium, followed by complete adynamia, fall of pulse which had been high, lessening of the body heat, difficulty of respiration, cutaneous anesthesia, presis, general and partial convulsions, retention, followed by incontinence of the urine and feces, weakened and irregular pulse and finally death from asphyxia in from three to thirty-six hours. Large doses paralyze the muscular motor nerve-ends and the cutaneous sensory nerve-ends, and hypodermic injections cause more local anesthesia than do those of morphine. Full doses destroy the tactile sense and lower muscular power. Following small doses there is a paralysis of the intracardiac vagus ends, with an enormous rise of the pulse-rate. It probably has some other action on the heart, as it relieves the paralysis following the administration of certain other drugs. Atropine acts upon the secretory nerve fibers and interferes with, or stops, glandular action, and through its action upon the motor nerve-centers arrests peristalsis of the intestines, as well as the peristaltic and contractive action of other organs. It causes a dilatation of the peripheral blood-vessels, especially those of the head and trunk, followed by a decided redness of the surfaces. This is also due to the increased pulse-rate and rise of the blood-pressure. Atropine exerts an antiphlogistic effect upon the vaso-motor nerves, with dilatation of the arteries, acceleration of the venous circulation, but with no appreciable effect upon the capillaries. It checks the passage of white globules into the tissues and causes a loss of movement of the emigrating cells. Following atropine there is a lessening of the number of leucocytes and the excretion of uric acid. Doses of one milligramme, given three times a day, effect the eye, the glandular innervation and organs containing unstriped muscle-fiber. Atropine either checks, or completely overcomes, the action of all of the salivary glands, stops the action of the mucous glands of the tongue, and lessens the sense of smell by drying of the nasal mucosa. It exerts less energy upon the glands of the digestive apparatus, although it checks the secretion of pepsin and HCl and of the pancreatic secretions. It overcomes the action of certain irritations and drug actions which increase pancreatic action. Atropine lessens the excretion of urine and urea, checks perspiration and possibly lessens the secretion of milk and lowers the secretion of the respiratory mucosa. Not all of the organs are affected in like manner, as the bladder may retain its activity, even after paralysis of the oesophagus. The action upon the circular muscle-fibers of the blood-vessels is both local and from the vaso-motor centers. There is first a brief slowing of the circulation, followed by pronounced acceleration, due to the relaxation of the circular fibers. Small doses paralyze cardiac inhibition, with increase of pulse-rate. By paralysis of the vagus ends, atropine slows the breathing and, through nerve-center action, prolongs and deepens the respirations. Atropine is a brain irritant, large doses causing excitement, unrest, insomnia, haste in movement and combativeness. Full intoxicant doses are followed by hallucinations of sight and hearing, delirium mostly cheerful, erotism, impulse to constant activity and finally convulsions, followed by paretic symptoms, anesthesia, syncope, anesthesia, sopor and very large doses paralyze striated muscle. There is a rise of temperature, and both heat formation and heat radiation are increased, this being due to the action directly upon the heat center of the brain. Some tolerance for the drug may be acquired, but cumulate action is very liable to occur.

The synergists are hydrocyanic acid, quinine, cicutine, morphine, strychnine, camphor monobromide, gelsemin, cypripedin, camphor, capsicin and glonoin, while the antagonists are nicotine, muscarine, pilocarpine, apomorphine, physostigmine, gelsemin, cicutine, the bromides and morphine. Adults do not bear atropine as well as do children.

Because of its mydriatic action, atropine is employed very largely by the oculists and is indispensable in their practice and is employed for the purpose of dilatation of the pupil prior to examination of the eye. It is indicated in superficial inflammations of the cornea with photophobia, especially if complicated with maladies of the iris, but is contraindicated in deep ulcerations of the cornea threatening perforation and conditions with increased intraocular pressure. In inflammation of the iris, adhesions may be prevented by the use of atropine. Local application to the conjunctiva may give relief in the headache due to eye-strain. Instillations give relief in immature cataract and spasm of accommodation.

Internally, atropine is a valuable remedy in painful affections of the skin, mucous membranes and muscles. Because of its action to relax muscular contraction, atropine is indicated in spasmodic conditions such as constriction of the sphincters, spasm of smooth muscle-fibers, oesophagismus, cardialgia, intestinal colics, hepatic, renal and uterine colics, and spasms of the neck of the bladder, of the uterus, vaginal and urethral orifices and of the sphincters of the anus. It is indicated in pathologic contractions or excessive activity of the non-striated muscles and consequently is useful in tenesmus of dysentery, in nocturnal enuresis, in lead colic, obstruction, in strangulated hernia, and has been used to good effect where fecal vomiting has been present. It is useful in persistent constipation. It prevents spasm in whooping cough, spasmodic asthma and all irritative coughs. Atropine is indicated in all cases of excessive secretion and is useful in the night-sweats of phthisis, salivation, bronchorrhea and diarrhea. Through antagonizing vagus irritation it is useful in the treatment of Asiatic cholera and other choleric bowel affections. It has been found of value in the treatment of various neuralgias and may be employed to advantage in epilepsy not due to sexual excitation, and has been found useful in eclampsia and hysteric spasms. Following glonoin in epileptic spasm, it prolongs the action of that drug by continuing the dilatation of the cutaneous blood-vessels and relieving the pressure to the brain. Used in full dosage during the early stages of acute catarrhs, it frequently brings about permanent relief. Atropine is being employed largely instead of morphine in the treatment of many painful affections, and with good results.

Atropine, through the dilatation of the peripheral vessels, acts as a powerful hemostatic, and is indicated in all hemorrhages. To obtain such relief, the drug should be administered in full dose, hypodermically, sufficient to produce a pronounced flushing of the skin, thereby withdrawing the blood from the site of the hemorrhage. The employment of the drug in this connection is one that should never be overlooked, as its prompt employment may result in the relief of what might have been a fatal termination. Atropine is indicated in cases of collapse with contracted pupils, cold surface covered with a cold clammy sweat and cold extremities, and more or less spasm, all indicative of internal congestion. In such cases a full dose, hypodermically, brings the blood to the surface and brings about an equalization of the circulation. Atropine gives relief in cerebral inflammation with retarded pulse and is useful in eruptive fevers with delayed eruptions and nervous symptoms attendant thereto. The specific applications of atropine are legion and it is a remedy worthy of much study clinically.

The recommended dose is 1-1000 grain, given at intervals of from five to twenty minutes, according to the urgency of the case and until there is dryness of the mouth, at which time it should be either withdrawn or the intervals increased, if it is desirable to maintain this effect. Should the drug not give the desired action when this effect has obtained, other remedy or surgical interference is indicated. In cases of hemorrhage or collapse, full doses should be administered hypodermically, in that the immediate physiologic action may be obtained. The dose in such instances is from 1-100 to 1-50 grain of the sulphate.

---

For Texas Medical Journal.

